# Digital and remote behavioral therapies for treating tic disorders: Recent advances and next steps

**DOI:** 10.3389/fpsyt.2022.928487

**Published:** 2022-07-15

**Authors:** Kareem Khan, Chris Hollis, Tara Murphy, Charlotte L. Hall

**Affiliations:** ^1^NIHR MindTech MedTech Co-operative, Institute of Mental Health, School of Medicine, Mental Health and Clinical Neurosciences, University of Nottingham, Nottingham, United Kingdom; ^2^NIHR Nottingham Biomedical Research Centre, Institute of Mental Health, Mental Health and Clinical Neurosciences, University of Nottingham, Nottingham, United Kingdom; ^3^Mental Health and Clinical Neurosciences, School of Medicine, University of Nottingham, Nottingham, United Kingdom; ^4^UCL Great Ormond Street Institute of Child Health (ICH), London, United Kingdom; ^5^Psychological and Mental Health Services, Great Ormond Street Hospital for Children NHS Foundation Trust, London, United Kingdom

**Keywords:** tics, Tourette syndrome, review, digital interventions, behavioral therapy, treatment

## Abstract

The rapid expansion of access to and engagement with digital technology over the past 15 years has transformed the potential for remote delivery of evidence-based digital health interventions (DHIs). Digital and remote behavioral interventions have the potential to address current gaps in the provision of evidence-based therapies in healthcare services. As the lack of access to behavioral treatments for people with tic disorders is a pressing issue across the world, there is great potential for DHIs to close this treatment gap. Here, we present a critical synthesis of the recent key advances in the field of digitally delivered, remote therapy for tics, outlining the research evidence for the clinical and cost-effectiveness and acceptability of digital or remotely delivered therapy. We found five trials aimed at reducing tic severity in children and young people and one trial for adults. The evidence supports the clinical utility of DHIs to deliver tic therapies, which shows promise in being clinically efficacious compared to an active control. Furthermore, DHIs in trials show good adherence and engagement and are acceptable to patients. The role of human support (including therapists and parents for young people) is likely to be important to encourage adherence. DHIs, where the main therapeutic content is delivered *via* web-based chapters, are likely to reduce clinical time, and maintain intervention fidelity, but further research is required to understand cost-effectiveness. Despite utilizing randomized controlled trials, only two trials were sufficiently powered to address efficacy and only one trial explored contextual factors that may influence engagement. Moreover, only one trial followed patients for >12 months, thus further long-term follow-ups are required. Specifically, we note that despite an emerging evidence base, DHIs for tics are yet to be routinely implemented in healthcare provision in any country. Drawing on the existing evidence, we conclude by proposing a stepped care model, in which digital therapy is implemented as a widely accessible first-line treatment using a purely online or therapist-supported approach.

## Introduction

Tic disorders, such as Tourette syndrome (TS), affect around 1% of children (1) and around 0.05% of adults (2) and are associated with a range of co-occurring behavioral, motor, and emotional conditions which can have a profound impact on children's and adult's quality of life, school/work experience and peer relationships (3). Although pharmacological interventions can be useful for people with tic disorders, behavioral and educational approaches are generally recommended in guidelines as a first line intervention (4). However, access to evidence-based behavioral therapies is limited due to the small number of highly trained therapists based in a few specialist centers with an uneven geographical distribution of services relative to demand. Digital health interventions (DHIs) provide the opportunity to widen access to psychoeducation and evidence-based behavioral therapies and thus reduce the severity and impact of debilitating conditions such as tic disorders.

Although studies have shown that DHIs can be efficacious in reducing symptom severity in people with tic disorders (5), no DHIs for tic disorders have yet to be implemented into routine clinical care. Digital delivery encompasses different types of treatment with varying active ingredients. The treatments are based on established techniques including Habit Reversal Therapy (HRT), in which patients learn to detect tics and use a competing response (usually an incompatible action) to control them; Comprehensive Behavioral Intervention for Tics (CBIT), which combines HRT with relaxation, functional analysis, and social support; Exposure and Response Prevention (ERP), in which patients learn to suppress their tics (response prevention) while tolerating urges to tic (exposure); and psychoeducation, where the focus is on the history, prevalence, and risk typically associated with tic disorders, and advice on healthy habits but with no information on tic control. Here, we review the recent key advances in the field of digitally delivered and remote therapy for tic disorders, outlining the research evidence for the clinical efficacy and cost-effectiveness and acceptability of these therapies. Efficacy refers to evidence gathered within tightly controlled trials whereas effectiveness refers to trials conducted in real-world settings. We explore strengths and limitations in the research design as well as investigating differences in the therapeutic approaches (i.e., type of therapy, use of blended human support, mode of delivery) of research to date. In doing so, we outline gaps for future research; examine the importance of the human factor in digital modalities, and implications for future care pathways as well as recommendations for practice. This paper examines the evidence for the efficacy of DHIs for tic disorders, which can be used to inform future research looking into the effectiveness of these interventions in order to assess the potential for implementation.

## Overview of digital and remote therapies

Recent advances in the use of digital technology have coincided with increasing rates of mental health and behavioral problems in young people and a growing demand for mental health services that outstrips supply and the capacity of traditional therapeutic approaches to respond. Thus, health services are turning to digital modalities to reach a larger proportion of the population (e.g., people who may be under provided for by standard face-to-face care) in a more efficient and patient-centered manner. DHIs refer to interventions delivered v*ia* technologies using a range of digital modalities, such as smartphones, applications (“apps”), wearable devices, robotics, websites, social media, or text messaging. DHIs can be used as a platform to help treat a range of physical and psychiatric disorders (6) promote positive health behaviors (7) and even improve outcomes of people with long term conditions (8). There is considerable optimism within the medical community that digital technologies–especially apps used on smartphones, tablets, and watches–could open a new frontier for the implementation of interventions to aid in the recovery from a range of disorders (9). Despite there being an estimated 350,000 health apps available to download across the major app stores (10), the vast majority have little or no evidence base.

These digital interventions may be delivered with varying degrees of human support. On one end of the spectrum, the intervention is delivered in a purely self-directed manner, with no therapist or human support. On the other end of the spectrum, the technology may be simply used as a vehicle for a therapist to remotely deliver therapeutic content in real-time (such as cognitive behavioral therapy delivered *via* videoconferencing). In the middle, there is a more “blended” approach whereby the technology platform is used to deliver the core therapeutic content with therapist support. This support may be provided synchronously or asynchronously (i.e., immediate or delayed responses) and be limited to only motivational or trouble-shooting advice or provide an adjunct to the therapeutic content (11).

For this review, we performed a non-systematic literature search using key terms such as digital interventions and tic disorders in databases including PsychINFO, PubMed, Embase, Central, Web of Science, and Medline. We also consulted with our clinical expert team to see if we omitted any studies of relevance. Studies were selected if the intervention aimed to improve the diagnostic symptomology of the tic disorder and was delivered *via* a website, mobile application (“app”), social media, email, or other form of digital technology. The intervention could include human support in its delivery and there was no restriction on targeted age. The search resulted in six trials for review.

## Videoconference delivered therapy for tic disorders

Initially, DHIs could only be delivered through desktop computers either locally or *via* modem connectivity meaning that users needed to be in a specific location to access the intervention. Indeed, the first two studies using digital modalities to deliver therapeutic content to people with tic disorders used videoconferencing software (“Skype”). Himle et al. (12) carried out the first pilot randomized controlled trial (RCT) within the realm of digital therapy for tic disorders (see Table 1 for summary of included studies). Extending on the findings of a previous pilot trial (13), they compared videoconferencing delivered CBIT to face-to-face CBIT for 8–17-year-olds with tics in USA. Participants (*N* = 20) attended 8 weekly sessions of CBIT at one of two university-based tic disorder specialty clinics over 10 weeks. Therapists were doctoral level psychologists with extensive CBIT training and experience, and study personnel were on hand to help participants connect to the remote therapist and to manage any technical difficulties. The primary outcome was tic severity as measured on the Yale Global Tic Severity Scale Total Tic Score (YGTSS-TTS) (14). The researchers found a statistically significant reduction in tic severity scores from baseline to 10-week follow-up (post-intervention) in both groups. Although the mean reduction in YGTSS-TSS in the videoconferencing group (7.8-point reduction) was greater than that of the face-to-face group (6.5-point reduction), this did not reach statistical significance between groups. Furthermore, positive treatment response as measured on the Clinical Global Impressions Improvement (CGI-I) (15) scale showed similar between group findings, with 80% being classified as treatment responders in the videoconferencing group compared to 75% in the face-to-face condition. This study indicated that videoconferencing was at least as efficacious as face-to-face therapy. Moreover, a measure of treatment credibility was similar between the two modes of delivery. Overall, this was the first RCT to show promising findings with regards to both positive outcomes and treatment acceptability in the domain of DHIs for tics.

**Table 1:** Summary of included studies.

**Reference**	**Design, number of arms, comparator, sample size and study location, setting**	**Sample demographics and baseline tic severity**	**Intervention and modality**	**Length/** **dosage, follow-ups**	**Comorbidities**	**Outcome measures**	**Human support with intervention**	**Adherence and engagement**	**Summary of main findings**
Himle, et al. (12)	RCT 2 arms, F2F CBIT, *N* = 20, USA, clinic	Children (8-17 yrs old, M = 11.6), 94% male, 28% on tic medication, 67% TS only, baseline YGTSS-TTS = 23.7	Internet-accessed Videoconference (Skype) CBIT	8 weekly sessions of CBIT delivered over 10 weeks. FU = post-treatment (week 10), and at 4-months	33% anxiety, 28% ADHD, 22% OCD	YGTSS^*^, CGI-S and CGI-I, PTQ, WAI, TAQ	Therapist supported	2 dropped out before primary analysis; both in F2F group	The intervention group showed a mean YGTSS-TTS reduction of 7.8 points and the F2F group showed a mean reduction of 6.5 points. Within-group ES for the two treatment delivery modalities were ES = 0.54 and ES = 0.75, for intervention and F2F. The intervention group showed a mean YGTSS-TTS reduction of 6.4 points at follow-up and the F2F group showed a mean reduction of 4.2 points. Within-group effect sizes for the two delivery modalities were ES = 0.39 and ES = 0.41, for intervention and F2F.
Ricketts et al. (16)	RCT 2 arms, WLC, N=20, USA, clinic and home based	Children (8-16 yrs old, M=12.1), 64.9% male, 95.8% Caucasian, 35% on tic medication, 75% TS only, baseline YGTSS-TTS = 25.75	Internet-accessed Videoconference (Skype) CBIT	Treatment consisted of two 1.5-h sessions followed by six 1-h sessions occurring over a 10-week period. FU = 10-week post treatment	25.8% ADHD, 8.3% OCD	YGTSS^*^, CGI-I, PTQ, CPTR, CSQ, TAQ, VSQ	Therapist and parent supported	Only 1 patient discontinued treatment as they sought treatment for OCD instead	In the intervention group there was a statistically significant decrease of 7.25 points in YGTSS-TTS total scores from baseline to post-assessment. In the WLC group, the 1.75-point decrease on the YGTSS-TTS total scores from baseline to post-assessment was not significant.
Andrén et al. (17)	Pilot RCT 2 arms, No comparison between groups, *N* = 23, Sweden, home based	Children (8-16 yrs old, M = 12.3), 65% male, 17.5% on tic medication, baseline YGTSS-TTS = 23.6	Internet delivered ERP and HRT	10 chapters over 10 weeks. FU = post-treatment and 3 (primary endpoint), 6 and 12-month	39% ADHD, 13% OCD	YGTSS^*^, CGAS, CGI-S and CGI-I, PUTS, GTS-QOL, adapted child version of the WSAS, OCI-Child version, CDI-S, PTQ, WSAS-Y (parent), SMFQ	Therapist and parent supported	Average number of completed chapters was 7.92 (for both children and parents) in the ERP group, and 7.36 (children) and 7.09 (parents) in the HRT group. 6 children (50%) and 5 parents (42%) in the ERP group, and 5 children and parents (45%) in the HRT group completed all 10 chapters. None lost to FU.	Significant reduction on the YGTSS-TTS for internet ERP, but not for internet HRT. Within-group Cohen's *d* was 1.12 for internet ERP and 0.50 for internet HRT.
Rachamim et al. (19)	Feasibility and effectiveness study with crossover design, 2 arms, WLC, N=41, Israel, home based	Children (7-18 yrs old, M = 11.26), 70.7% male, 24.4% on tic medication, baseline YGTSS-TTS = 22.72	Internet delivered CBIT	9 modules over 9 weeks. FU = post-treatment, 3 and 6-months	43.9% ADHD, 31.7% OCD	YGTSS^*^, CGI-I, CGAS, ADIS, PTQ, Revised CPRS, OCI, SCARED, LSAS, RSES, CDI	Therapist and parent supported	23 completed 9 modules. Participants completed a mean of 8.8/9 modules. Reasons for stopping (*n* = 2) included a lack of motivation and self-discipline.	A significant interaction was found for the YGTSS-TTS between time-point and group [*F*_(1, 39)_ = 9.96, *p* = 0.003, large effect]. At post-intervention (time 2), the YGTSS-TTS was significantly reduced in the internet CBIT arm only. Internet CBIT was associated with a mean YGTSS-TTS reduction of 6.60 points (*p* < 0.001) compared with a mean YGTSS-TTS reduction of 0.94 points (*p* = 0.51) in the WLC arm. This 6.60 points difference was clinically meaningful, with an ES of within-group Cohen's *d* = 0.91, large effect.
Hollis et al. (18)	RCT 2 arms, Internet Psychoeducation, *N* = 224, UK, home based	Children (9-17 yrs old, M = 12), 79% male, 87% White, 13% on medication for tics, baseline YGTSS-TTS = 28.4	Internet delivered ERP	10–12 weeks of 10 chapters for both child and parent. FU = 3-, 6-, 12- and 18-months post-randomization	27% anxiety disorder, 25.5% ADHD, 22.5% ODD	YGTSS^*^, CGI-I, CGAS, CASUS, CHU9D, SDQ, PTQ, modified version of the Hill and Taylor side-effects scale, MFQ, SCAS, PUTS, C&A-GTS-QOL	Therapist and parent supported	204 (91%) received the minimum intervention (at least first 4 chapters) and were treatment completers (99 in the ERP group and 105 in the psychoeducation group). 186 (83%) were followed up 6 months after randomization (93 in the ERP group and 93 in the psychoeducation group).	Mean total decrease in YGTSS-TTSS at 3 months was 4.5 (16%) in the ERP group vs. 1.6 (6%) in the psychoeducation group, and at 6 months was 6.9 (24%) in the ERP group vs. 3.4 (12%) in the psychoeducation group. The estimated mean difference in YGTSS-TTSS change between the groups at 3 months was −2.29 points (95% CI −3.86 to −0.71) in favor of ERP, with an ES of −0.31 (95% CI −0.52 to −0.10)
Haas et al. (20)	RCT 3 arms, Placebo and F2F CBIT, *N* = 161, Germany, home based	Adults (112 males, 49 females, mean age = 35.6 yrs old, range = 18–62 yrs), 40.4% on tic medication, baseline YGTSS-TTS = 24.37	Internet delivered CBIT	8 sessions over 10 weeks. FU = 5 weeks after start of treatment (V2), 1 week after end of treatment (V3; primary endpoint), and 2 follow-up visits at 3 (V4) and 6 months (V5)	Not reported	YGTSS^*^, Modified RVBTRS, Adult Tic Questionnaire, GTS-QoL, PUTS-9, CGI-S and CGI-I, Y-BOCS, Conners' Adult ADHD Rating Scales, BDI-II, BAI, WAI-SR	No human support	108 (67.1%) were considered as compliant until V3. Rate of non-compliance was lowest in the placebo group (22.9%) and similarly high in both treatment groups	Internet CBIT group showed a larger tic reduction [2.54 (−3.53; −1.55)] in comparison to the placebo group [−1.26 (−2.16; −0.35)] at V3. Difference in YGTSS-TTS change to baseline between placebo and internet CBIT was −1.28 (−2.58; 0.01). Significance for superiority of internet CBIT was narrowly missed and the null hypothesis could not be rejected as the upper 95% CI limit was marginally above 0. Difference in YGTSS-TTS change to baseline between internet CBIT and F2F CBIT at V3 was 0.98 [−1.01; 2.96]. Since the upper bound of the 95% CI was below the non-inferiority margin of 3; non-inferiority of internet CBIT in comparison to F2F CBIT could be observed.

* *Primary outcome measure. ADHD, attention deficit hyperactivity disorder; ADIS, Anxiety Disorders Interview Schedule; CBIT, Comprehensive Behavioral Intervention for Tics; CDI, Children's Depression Inventory; CGAS, The Children's Global Assessment Scale; CGI-I, Clinical Global Impression-Improvement Scale; CGI-S, The Clinical Global Impression-Severity scale; CHU9D, Child Health Utility instrument; CPRS, Child-Parent Relationship Scale; CPTR, Children's Perception of Therapeutic Relationship; CSQ, Client Satisfaction Questionnaire; ERP, Exposure and Response Prevention; ES, effect size; F2F, Face-to-face; FU, Follow-up; GTS-QOL, Gilles de la Tourette Syndrome-Quality of Life Scale; HRT, Habit Reversal Therapy; LSAS, Liebowitz Social Anxiety Scale; MFQ, Mood and Feelings Questionnaire; OCD, obsessive compulsive disorder; OCI, Obsessive-Compulsive Inventory; ODD, Oppositional defiant disorder; PTQ, Parent Tic Questionnaire; PUTS, Premonitory Urges for Tic Disorders Scale; RCT, randomized controlled trial; RSES, Rosenberg's Self-Esteem Scale; RVBTRS, Rush Video-Based Tic Rating Scale; SCARED, Screen for Child Anxiety Related Disorders; SCAS, Spence Children's Anxiety Scale; TAQ, Treatment Acceptability Questionnaire; TAU, Treatment as usual; TS, Tic syndrome; TTS, Total Tic Score; VSQ, Videoconferencing Satisfaction Questionnaire; WAI, Working Alliance Inventory; WLC, wait-list control; WSAS, Work and Social Adjustment Scale; YGTSS, Yale Global Tic Severity Scale*.

Following on from this study, Ricketts et al. (16) conducted a similar RCT also in USA, however, they compared videoconferencing CBIT to a waitlist control. Participants (*N* = 20) were 8–16-year-olds and therapeutic content was delivered by a therapist located in a university-based tic disorders specialty clinic. However, in contrast to Himle et al. (12), participants accessed videoconferencing therapy from home. Both the child and their parent were required to be present for sessions, although for mature older adolescents (i.e., those who were 16 years) this was waived and they could attend alone. Treatment consisted of two 1.5-h sessions followed by six 1-h sessions occurring over a 10-week period. Parents were urged to reward children to help their engagement rates and participants were financially rewarded by the study team for completion of both the baseline assessment and the post-assessment. The study found no statistically significant difference in tic severity scores between the videoconferencing and waitlist group. However, in the videoconferencing group there was a significant within-group reduction in tic severity scores between baseline and follow-up (10-weeks post baseline) which was not observed in the control group. Furthermore, there were a significantly higher proportion of treatment responders in the videoconferencing CBIT group (33.3%) relative to waitlist control (0%) and parent acceptability ratings were high. Given the small sample sizes, it is unlikely that the samples of either Himle et al. (12) or Ricketts et al. (16) were powered to detect the effect of DHIs on clinical outcomes, however, the findings provide preliminary support and acceptability of DHIs for tics.

## Web-based internet therapy for tic disorders

Whilst the two studies described showed promising findings and potentially opened a new frontier for delivering evidence-based treatments *via* digital modalities, either the participants and/or the therapists had to be present at a clinic for the sessions and the technology was used as a vehicle to aid remote human therapist delivery of the intervention: it doesn't address a critical factor affecting access which is the lack of highly trained therapists. As digital technology progressed exponentially in the years since the Ricketts et al. (16) study in 2016, there was a move away from videoconferencing to mobile, remote technology, which allowed more flexibility for participants to complete sessions at their own pace at a setting of their choosing. Moreover, smartphones could now be integrated to send SMS or emails as an adjunct to regular face-to-face therapy with therapeutic content delivered by web-based chapters. Andrén et al. (17) were the first to take advantage of this new technology in the tic disorder domain. They conducted a pilot RCT in Sweden evaluating two types of internet-delivered behavioral therapies: ERP and HRT. Participants (*N* = 23) were 8–16-year-olds who completed 10 web-based chapters of remote therapeutic content, similar to a self-help book, over 10 weeks with parental support. Parents had separate logins to the online platform and were able to access extended versions of the treatment content. Specifically, parents learnt about parental coping strategies, social support, and functional analysis (i.e., examining the causes and consequences of behavior). They also had access to a therapist who did not deliver any therapeutic content. Therapists were supervised, graduate psychologists who were trained in the use of the platform and were mainly responsible for engaging participants and responding to any queries *via* the online platform or SMS. Therapists answered queries *via* the online platform, which related to understanding treatment content delivered in the web-based chapters and any technical difficulties, but they did not provide any new treatment content relating to ERP/HRT. The researchers found that there was a significant reduction on the YGTSS-TTS for the ERP group, but not for the HRT group 3-months post-intervention. Within-group Cohen's *d* was 1.12 for ERP and 0.50 for HRT. In addition, 9 participants (75%) in the ERP group and 6 participants (55%) in the HRT group were classified as treatment responders according to the CGI-I and children and parents rated both treatments as credible and satisfaction at post-treatment was high in both groups. Adherence and engagement were excellent in both groups.

The Andrén et al. (17) study showed promising findings that tic severity can be reduced with the use of remote therapist supported internet delivered behavioral therapy, but the study was not powered to explore clinical efficacy. Hollis et al. (18) expanded on this pilot by conducting a large RCT in England using the same online platform as Andrén et al. (17) with the content translated into English language. In total, 224 participants aged between 9 and 17 years were randomized to receive either internet ERP or online delivered psychoeducation as an active control. ERP was chosen as the active therapeutic intervention based on the findings from Andrén et al. (17) which suggested that ERP may be more acceptable and feasible to deliver in an online format. Aligned with the pilot Swedish study, participants were required to work through 10 chapters of content over 10 weeks with parental and therapist support. Parents had their own chapter content to work through which gave them tools to help support their child during treatment as well as more information on tic disorders and related conditions. The therapists' role was to answer any queries and engage participants but not deliver any therapeutic content. The findings showed that at 3 months post-baseline there was significant reduction in tics in the ERP group (4.5, 16% YGTSS-TTS reduction) compared to the psychoeducation group (1.6, 6% YGTSS-TSS reduction). The estimated mean difference in YGTSS-TTS change between the groups at 3 months was −2.29 points in favor of ERP, with an effect size of −0.31 (95% CI −0.52 to −0.10). There was also a significantly greater positive treatment response with ERP at 3 months (36%) than with psychoeducation (20%). Adherence and engagement in both groups was excellent and the perception of treatment suitability, credibility and satisfaction was high across both groups. Although a full economic analysis is to be reported in the long-term follow-up Online Remote Behavioral Intervention for Tics (ORBIT) paper, preliminary analysis showed that the fixed and variable costs including wider healthcare costs of delivering the behavioral therapy (ERP) were higher compared to psychoeducation [£159 (95% CI 53–370) more per participant]. As the study did not compare to standard face-to-face therapy it is not possible to understand cost-savings compared to standard tic services. However, the authors indicate that given the total therapist time in the trial was an average of 2.5 h delivered by a less-experienced therapist compared to typically 9–10 h of highly skilled therapist time required for face-to-face therapy, it is possible this would be cost-effective. In sum, this was the first adequately powered RCT that showed internet delivered behavioral therapy with low intensity human support could reduce tic severity offering a new approach to breaking down barriers in accessing evidence-based treatments.

Two further trials have been conducted that evaluated remote digital behavioral therapies using a web-based delivery approach. One used a crossover design and was carried out in Israel by Rachamim et al. (19). They compared caregiver-guided self-help internet delivered CBIT to a waitlist control group in a sample of 41 children and adolescents (7–18 years). The therapeutic content was delivered *via* nine web-based chapters over 9 weeks, and participants had parental support with access to a therapist, who provided support but did not deliver any therapy. At post-intervention, the YGTSS-TTS was significantly reduced in the internet CBIT arm only with a mean YGTSS-TTS reduction of 6.60 points compared with a mean YGTSS-TTS reduction of 0.94 points in the waitlist arm. The 6.60 points difference had an effect size of within-group Cohen's *d* = 0.91, indicating large effect. All but one of the participants in the internet CBIT group (95%) were rated as treatment responders.

The final study was carried out by Haas et al. (20) in Germany and the sample was 161 adults. This was the only study in the literature conducted in an adult population. They compared self-directed internet CBIT delivered *via* web-based chapters to placebo and face-to-face CBIT, with participants completing 8 sessions over 10 weeks. The study found no significant difference in efficacy between web-based and face-to-face delivered CBIT (non-inferiority) and although the web-based CBIT group showed a larger tic reduction compared to the placebo condition, this fell short of statistical significance. Overall, these two studies further add to the promising findings that digital technology could be used to deliver evidence-based behavioral treatments to people with tic disorders.

## Strengths and limitations

In critically appraising the evidence of DHIs for people with tic disorders, one must consider the inherent strengths and limitations within the respective studies. All but one of the studies employed a randomized controlled design, which is considered the “gold standard” for efficacy studies. However, most were not sufficiently powered to address efficacy. All but two of the included trials had small sample sizes (i.e., <50), which means that studies were probably underpowered to reliably detect clinically meaningful effects. One intrinsic methodological limitation of many therapeutic intervention trials is the great difficulty in blinding participants and those delivering treatment (21), thus introducing a high risk of bias. This can be partially mitigated by having outcome assessors (such as YGTSS assessors) who are blind to arm allocation, which was done in all the presented trials.

Before any new technology can be implemented in routine practice, it is important to understand the costs of an intervention to the healthcare system. Economic evaluations can be used to inform decisions about the economic impact and relative value for money of DHIs. It can assess whether differences in costs between the intervention and competing alternatives can be justified in terms of health and non-health benefits. However, only one of the papers included a full health economic analysis (18). Moreover, only one of the included trials was conducted in an adult population and whilst the sex distribution in the included studies is typical for a tic disorder population, a large proportion of participants in the studies were white, which may limit the generalizability of the findings concerning ethnicity. Another criticism of the included studies is the lack of long-term follow up data. It is imperative to understand the sustainability of digital interventions, however most of the included trials were of limited follow up with only one of the trials measuring outcomes beyond 12-months (18, 22).

Furthermore, there is an issue with generalizing the findings to routine practice. As a small proportion of participants in the included trials had comorbidities, this may not reflect the reality of standard practice especially as research suggests that around 85–88% children with tic disorders have at least one psychiatric comorbidity (23). The reported studies incorporated a range of therapeutic content and approaches, differed in their level of human involvement, and had varied comparators and modalities of delivery, which could have affected participant interaction and consequently, efficacy (24). Further research is required to understand better as to what works best and for whom.

Despite these limitations, the included studies reported promising findings that give cause for optimism in utilizing digital technology for people with tic disorders. First, all participants who received the digital treatments in the respective studies showed some improvement in tic severity from baseline to primary endpoint as measured on the YGTSS-TTS, which ranged in a mean reduction of 4.5 points in Hollis et al. (18) to 7.8 points in Himle et al. (12). Although these reductions were over a similar timeframe, the Hollis et al. (16) study had a far larger sample size which may explain the discrepancy in tic reduction between the two studies. Furthermore, a larger proportion of those who received a digital intervention showed positive treatment response compared to controls. The effect sizes, tic reduction, and responder statuses of included studies are comparable to previous studies assessing face-to-face therapeutic interventions for tic disorders (25, 26). Another positive outcome, which was found in the ORBIT trial, is that digital ERP could be delivered with around one quarter of the therapist contact time (also at a lower level of training) compared to evidence-based face-to-face behavioral therapy. Therapists required limited training in how to use the ORBIT platform and support the intervention. These are positive findings as they show that digitally enabled behavioral therapy has similar efficacy but lower costs than regular face-to-face therapy, and, if delivered as a first-line behavioral intervention, could allow more people to access evidence-based non-pharmacological interventions. Another strength of the trials is that they all used a validated and reliable measure, namely the YGTSS. As the YGTSS is a subjective, clinician-rated measure, it is imperative that researchers are trained and supervised throughout the trial in how to conduct this measure. Indeed, four out of the six trials included in this review explicitly mentioned training their YGTSS assessors.

Aside from efficacy, before any new intervention can be adopted in routine healthcare, assessments must be made on how acceptable and/or credible participants found it. This is particularly important when evaluating modern advancements such as digital therapies. Indeed, all included studies showed that participants were highly satisfied with the treatments and found the mode of delivery acceptable/credible. Another consideration is the extent to which the intervention was safe to deliver and use, which is generally captured in the form of adverse event reporting. All but two studies explicitly recorded and reported on adverse events. Although a few serious adverse events in total were reported across the included studies, none were related to the treatment, suggesting that all interventions were safe to use. Finally, all trials had low attrition and high engagement rates with the intervention. As high attrition and low engagement rates are a common problem in digital health research (27, 28), this not only shows the need that this population have for an evidence based behavioral treatment but that the included interventions appeared to be engaging to users. However, it is worth noting that all but one of the studies involved human support which may have positively impacted on engagement rates.

## Future research for tic-related digital interventions

Primarily, it would be important for any future work to supplement the limitations highlighted above. Only one of the included studies assessed the cost-effectiveness of the digital intervention, which is likely to be an important consideration for policymakers. A cost-effectiveness evaluation would be much needed in future research of digital interventions for tic disorders to help policymakers make decisions on adoption to routine healthcare. All but one of the interventions in this review contained an element of human interaction, either with synchronous contact by videoconferencing or asynchronous contact through SMS or the online platform. The best improvement in outcomes, therefore, may be achieved through a blended approach of online intervention and human support. As technology evolves rapidly, future online interventions will be more dynamic, perhaps including real-time therapist input and integrated synchronous crisis support. A promising new development is the use of virtual reality, which has had positive results on children with other neurodevelopmental disorders (29) and a range of other mental health problems (30) but has yet to be explored with individuals with tics. Developers could utilize virtual reality to its full effect and enable a simulated, life-like human therapist to support patients with tics, which would also be more cost effective than a human therapist an area worthy of future pursuit.

Future studies of digital interventions for people with tic disorders must have larger sample sizes to generate greater statistical power and allow for an increase in generalizability. Moreover, there should be a more concerted effort to diversify the inclusion criteria so that the samples are representative of clinical practice. They must also consider including long-term follow-up assessments to evaluate whether effects are maintained over a prolonged period. Only one of the included trials followed up participants beyond 12-months post-randomization (18, 22). Although currently under investigated, a potential strength of digital interventions is the delivery of treatment in geographically distant and economically challenged contexts, such as low- and middle-income countries, where knowledge and application of treatments is reported to be low (31). This area requires further research to define the barriers and benefits. Furthermore, as is known within the digital literature, it is crucial to understand how these complex interventions work and for whom. Thus, future RCTs evaluating DHIs for people with tic disorders should consider conducting a mixed methods process evaluation concurrently with trial delivery, as this would be useful in addressing the intervention's implementation, mechanisms of impact and context. Such findings were crucial in understanding the extent to which ORBIT was both implemented with a high degree of quality (32) and the mechanisms through which it achieved impact (33).

Despite much talk of triggering a revolution in health service delivery and treatment, digital interventions are rarely mainstreamed or sustained (34). This is partly because once a DHI has shown efficacy in an RCT, there is an unclear pathway to implementation. Therefore, the critical next step is to conduct a real-world implementation study to show proof-of-concept of a DHI for children with tic disorders. This could take the form of a process evaluation, effectiveness, and cost-effectiveness study. Furthermore, it would be sensible for any future real-world evaluation to employ an evidence-based implementation science framework to inform planning and evaluation. For instance, the NASSS model (Non-adoption, Abandonment, Scale-up, Spread and Sustain) (35) is a mixed-methods approach that considers the influence on implementation of complexities in key domains, such as target problem, technology, adopters, organization, and broader systems. This will enable policymakers to make decisions on strategies to reduce or address complexity, which may increase the likelihood of effective implementation and adoption in routine healthcare services.

Another promising route to implementation for digital interventions for tic disorders are hybrid implementation-effectiveness trials, which have the potential to be an appropriate design for simultaneously examining clinical and implementation outcomes for DHIs. This would save valuable time, as it would not rely on researchers carrying out efficacy trials entirely separately from implementation research. For example, Lane-Fall et al. (36) have developed a “subway line” of translational research that may be a helpful heuristic for conceptualizing future directions for hybrid implementation-effectiveness trials within the tic-related field. However, this requires a defined care pathway so the routes to accessing these treatments are clear, as that is often a significant barrier. For instance, these interventions would need to be overseen by a clinician with tic experience and knowledge, as it would not fit in with general practitioner's (GP) who do not necessarily have the expertise to deal with tic disorders.

## The human factor

One could argue that there is no need for a therapist and, to cut costs, all these digital therapies could be implemented as self-help programs; however, there is no empirical evidence to support this notion. Moreover, the literature suggests that supported digital interventions are more engaging and efficacious than non-supported interventions (5, 37, 38). Optimizing user experience, which is defined as the extent to which an intervention is perceived by a person as useful, enjoyable and user friendly, has great potential to address barriers to successful future implementation (39). Increasingly, human-centered designs are being employed in healthcare innovations to enhance user experience, thereby promoting better adherence and efficacy (40). As the adherence and engagement rates were high in all included studies, it is clear that human support played a crucial role in improving user involvement.

Another point of consideration that is specific for children and young people is the extent to which parents or carers should be involved. Most of the interventions in this review had some form of parental involvement, however the level of involvement differed between studies. It does seem that parents play a crucial role in engaging and ensuring the adherence of treatment content within these interventions. Indeed, the ORBIT trial's process evaluation found that parental engagement significantly influenced child's level of engagement (32) and efficacy (33). Several systematic reviews have also noted the crucial role parents have in positive outcomes for children and adolescents across a range of treatments for a variety of conditions (41–43). Parents bring a strong level of commitment, availability and personal expertise of their child that is an invaluable asset to researchers and clinicians so must be utilized in any future roll out. However, it must be noted that not all caregivers have the capacity to assist with the delivery of such interventions given systemic factors and competing demands. Therefore, there may also be value in designing interventions that can be delivered to children and young people whose parents do not have the capacity to engage regularly in treatment. Furthermore, it is worth considering that digital interventions have the potential to provide more equitable access to care for caregivers who have limited capacity to engage in face-to-face interventions (i.e., due to costs, travel, work schedules, busy lives).

## Recommendations for future practice

Face-to-face behavioral therapy is an effective treatment for tic disorders in children and adults, however less than one in five have access in the UK (44). Rates vary across the world but access to non-pharmacological treatments for tic disorders is low in many contexts, even those with good provision of care in other areas of mental health. All the studies in this review show that digital delivery of behavior therapy for tics can be an efficacious, engaging, and safe form of treatment. This could greatly increase access to therapy. With recent European clinical guidelines stating that behavior therapy should be offered as first line treatment option (4), it seems that the digital revolution offers a significant approach in overcoming the lack of access.

Despite this, there is a need to determine the optimum care pathways with respect to sequencing and integration of digital and face-to-face behavioral therapy for tics. For example, a stepped-care approach could be implemented whereby digital therapy is offered first, followed by more intensive face-to-face therapy for those who may require it. Initially, this should be offered to children and adolescents with tic disorders, as the research to date is less robust in adults with tics. Only one study in this review was conducted on adults with tics (20) and thus more research is needed to establish its efficacy before wider implementation.

In terms of what active components may be essential and what this digital therapy may look like in any future roll out in clinical services; this review may be able to shed some light on this. Firstly, based on the available evidence, it appears that either CBIT or ERP are likely to lend themselves to remote delivery. Although CBIT has the largest evidence base of any behavioral therapy in the tic literature (26), ERP is arguably more efficient and less intensive as a digital therapy. For instance, findings from the ORBIT trial, which used ERP as its form of therapy, showed that participants only required their therapists support for around 15 min per week and largely undertook the ERP practices themselves (18, 32). Moreover, therapists involved in the ORBIT study needed very little training and were less experienced than those who may deliver CBIT. Employing therapists with little experience and who are less qualified than a licensed doctoral-level therapist, for example, would also present better value for money for healthcare services, as they could be employed at a lower salary rate. However, caution must be taken with these considerations as none of the studies in this review included a comparison of which intervention and components are best delivered digitally.

Design considerations of DHIs are one of many factors that must be examined before any potential implementation. Firstly, it is essential to include patient and public involvement (PPI) in the process of designing and developing such interventions, as is consistent with user-centered design principles. Such insights from the PPI group involved in the ORBIT trial were pivotal to its successful recruitment and retention of participants (45). Findings from the literature suggest that individuals make credibility judgements about online information (46) and cost-benefit analysis of behavior (47) to determine their projections of continuing, especially in the early stages of treatment. Thus, it appears that developers of future iterations of DHIs for children with tic disorders must consider how to make these engaging and stimulating to facilitate continued usage. This may constitute specific features such as video demonstrations of therapy, animations, the ability to visualize which tics are increasing or decreasing in severity and frequency which may be especially engaging and enjoyable for children. Indeed, these interactive components were identified as key features of the ORBIT intervention and seemed to be used most (32). This is consistent with evidence that interactive elements, including attractive audio-visual material to be amongst the most highly used features of DHIs as they tend to keep users' interest (48, 49). This would be especially important to younger children whose concentration levels would not be maintained with material that was simply presented in writing, for example. It may also be sensible to include some sort of reward system. This seems to be an effective strategy to engage children and ensure that they maintain their level of commitment with the practices involved in behavioral therapy for tics.

## Conclusion

The available evidence indicates that DHIs have potential to be clinically efficacious in reducing tics as well as being acceptable to patients. Further research is required to determine cost-effectiveness. However, given potential cost-savings and service efficiencies associated with a release of clinical time, it is likely this would be cost-effective. Furthermore, additional research is needed to establish long-term impact and determine DHI in routine care pathways, outside of clinical trials. As all the research to date in this domain have been conducted in tightly controlled and monitored trials, the focus has been on efficacy rather than effectiveness. Digital technology evolves at a rapid pace meaning that as technology changes and interfaces are updated it cannot be certain that a program that was efficacious five or ten years ago would be equally efficacious today. Although RCTs are still the gold standard for which to assess the efficacy of DHIs, they can take many years to establish evidence meaning technology outpaces this. Thus, there is a need for more real-world evaluations to establish effectiveness. A digital intervention that could be deployed to large numbers of patients at a relatively low cost is a much needed and seemingly acceptable means of providing patients with access to evidence-based treatments. It could provide immediate access to these treatments for those who otherwise would not have access due to long waiting lists or their geographical location, which could also potentially free up existing resources and services for those requiring more complex treatment and assessment. Thus, cutting costs and waiting times would be a two-fold benefit for healthcare services and patients alike. There is a need to conduct more robust research in this domain but also an urgency to implement a digital intervention for children with tic disorders in real-world settings.

## Author contributions

KK and CLH outlined the structure, reviewed the literature, and wrote this paper. TM and CH provided critical feedback. All authors contributed to the article and approved the submitted version.

## Funding

This research was funded by the NIHR Health Technology Assessment (HTA) (Ref 16/19/02). This work was supported by NIHR MindTech MedTech Co-operative and MRC Digital Youth Programme grant.

## Conflict of interest

The authors declare that the research was conducted in the absence of any commercial or financial relationships that could be construed as a potential conflict of interest.

## Publisher's note

All claims expressed in this article are solely those of the authors and do not necessarily represent those of their affiliated organizations, or those of the publisher, the editors and the reviewers. Any product that may be evaluated in this article, or claim that may be made by its manufacturer, is not guaranteed or endorsed by the publisher.

## Author disclaimer

The views expressed are those of the author(s) and not necessarily those of the NHS, the NIHR or the Department of Health.

## References

[1] Scahill L Specht M Page C. The prevalence of tic disorders and clinical characteristics in children. J Obsessive Compuls Relat Disord. (2014) 3:394–400. 10.1016/j.jocrd.2014.06.002 25436183 PMC4243175

[2] Knight T Steeves T Day L Lowerison M Jette N Pringsheim T. Prevalence of tic disorders: a systematic review and meta-analysis. Pediatr Neurol. (2012) 47:77–90. 10.1016/j.pediatrneurol.2012.05.002 22759682

[3] Eapen V Cavanna AE Robertson MM. Comorbidities, social impact, and quality of life in tourette syndrome. Front Psychiatry. (2016) 7:97. 10.3389/fpsyt.2016.00097 27375503 PMC4893483

[4] Andrén P Jakubovski E Murphy TL Woitecki K Tarnok Z Zimmerman-Brenner S. European clinical guidelines for Tourette syndrome and other tic disorders—version 2.0. part II: psychological interventions. Eur Child Adolesc Psychiatry. (2021) 31:403–23. 10.1007/s00787-021-01845-z 34313861 PMC8314030

[5] Khan K Hall CL Davies EB Hollis C Glazebrook C. The effectiveness of web-based interventions delivered to children and young people with neurodevelopmental disorders: systematic review and meta-analysis. J Med Internet Res. (2019) 21:e13478. 10.2196/preprints.13478 31682573 PMC6858614

[6] Andersson G Cuijpers P Carlbring P Riper H Hedman E. Guided Internet-based vs. face-to-face cognitive behavior therapy for psychiatric and somatic disorders: a systematic review and meta-analysis. World Psychiatry. (2014) 13:288–95. 10.1002/wps.20151 25273302 PMC4219070

[7] Free C Knight R Robertson S Whittaker R Edwards P Zhou W. Smoking cessation support delivered via mobile phone text messaging (txt2stop): a single-blind, randomised trial. Lancet. (2011) 378:49–55. 10.1016/S0140-6736(11)60701-0 21722952 PMC3143315

[8] Murray E Burns J See TS Lai R Nazareth I. Interactive health communication applications for people with chronic disease. Cochrane Database Syst Rev. (2005) 4:Cd004274. 10.1002/14651858.CD004274.pub4 16235356 PMC13184810

[9] Ventola CL. Social media and health care professionals: benefits, risks, and best practices. P T. (2014) 39:491–520. 25083128 PMC4103576

[10] Research2Guidance. 325,000 Mobile Health Apps Available in 2017 (2017). Available online at: https://research2guidance.com/325000-mobile-health-apps-available-in-2017/ (accessed July 15, 2019).

[11] Sanderson C Kouzoupi N Hall CL. Technology Matters: The human touch in a digital age – a blended approach in mental healthcare delivery with children and young people. Child Adolesc Ment Health. (2020) 25:120–2. 10.1111/camh.12385 32307846

[12] Himle MB Freitag M Walther M Franklin SA Ely L Woods DW. randomized pilot trial comparing videoconference versus face-to-face delivery of behavior therapy for childhood tic disorders. Behav Res Ther. (2012) 50:565–70. 10.1016/j.brat.2012.05.009 22743661

[13] Himle MB Olufs E Himle J Tucker BTP Woods DW. Behavior therapy for tics via videoconference delivery: an initial pilot test in children. Cogn Behav Pract. (2010) 17:329–37. 10.1016/j.cbpra.2010.02.006

[14] Leckman JF Riddle MA Hardin MT Ort SI Swartz KL Stevenson J. The yale global tic severity scale: initial testing of a clinician-rated scale of tic severity. J Am Acad Child Adolesc Psychiatry. (1989) 28:566–73. 10.1097/00004583-198907000-00015 2768151

[15] Guy W National National Institute of Mental H. ECDEU assessment manual for psychopharmacology. DHEW publication; no. (ADM) 76–338. Rockville, Md.: U.S. Dept. of Health, Education, and Welfare, Public Health Service, Alcohol, Drug Abuse, and Mental Health Administration, National Institute of Mental Health, Psychopharmacology Research Branch, Division of Extramural Research Programs (1976). p. 603. Available online at: file://catalog.hathitrust.org/Record/101681632 http://hdl.handle.net/2027/uc1.31210000126621

[16] Ricketts EJ Goetz AR Capriotti MR Bauer CC Brei NG Himle MB. A randomized waitlist-controlled pilot trial of voice over Internet protocol-delivered behavior therapy for youth with chronic tic disorders. J Telemed Telecare. (2016) 22:153–62. 10.1177/1357633X15593192 26169350 PMC6033263

[17] Andrén P Aspvall K Fernández de la Cruz L Wiktor P Romano S Andersson E. Therapist-guided and parent-guided internet-delivered behaviour therapy for paediatric Tourette's disorder: a pilot randomised controlled trial with long-term follow-up. BMJ Open. (2019) 9:e024685. 10.1136/bmjopen-2018-024685 30772854 PMC6398666

[18] Hollis C Hall CL Jones R Marston L Novere Le M Hunter R. Therapist-supported online remote behavioural intervention for tics in children and adolescents in England (ORBIT): a multicentre, parallel group, single-blind, randomised controlled trial. Lancet Psychiatry. (2021) 8:871–82. 10.1016/S2215-0366(21)00235-2 34480868 PMC8460453

[19] Rachamim L Zimmerman-Brenner S Rachamim O Mualem H Zingboim N Rotstein M. Internet-based guided self-help comprehensive behavioral intervention for tics (ICBIT) for youth with tic disorders: a feasibility and effectiveness study with 6 month-follow-up. Eur Child Adolesc Psychiatry. (2020) 1:3. 10.1007/s00787-020-01686-2 33231786 PMC7683326

[20] Haas M Jakubovski E Kunert K Fremer C Buddensiek N Häckl S. ONLINE-TICS: internet-delivered behavioral treatment for patients with chronic tic disorders. J Clin Med. (2022) 11:250. 10.3390/jcm11010250 35011989 PMC8745756

[21] Baumeister H Hutter N Bengel J. Psychological and pharmacological interventions for depression in patients with diabetes mellitus and depression. Cochrane Database Syst Rev. (2012) 12:CD008381. 10.1002/14651858.CD008381.pub2 23235661 PMC11972844

[22] Hall CL Davies EB Andrén P Murphy T Bennett S Brown BJ. Investigating a therapist-guided, parent-assisted remote digital behavioural intervention for tics in children and adolescents—‘online remote behavioural intervention for Tics' (ORBIT) trial: protocol of an internal pilot study and single-blind randomised. BMJ Open. (2019) 9:e027583. 10.1136/bmjopen-2018-027583 30610027 PMC6326281

[23] Ueda K Black KJ. A comprehensive review of tic disorders in children. J Clin Med. (2021) 10:2479. 10.3390/jcm10112479 34204991 PMC8199885

[24] Gulliver A Griffiths KM Christensen H Brewer JL. A systematic review of help-seeking interventions for depression, anxiety and general psychological distress. BMC Psychiatry. (2012) 12:81. 10.1186/1471-244X-12-81 22799879 PMC3464688

[25] Cook CR Blacher J. Evidence-based psychosocial treatments for tic disorders. Clin Psychol Sci Pract. (2007) 14:252–67. 10.1111/j.1468-2850.2007.00085.x

[26] Whittington C Pennant M Kendall T Glazebrook C Trayner P Groom M. Practitioner review: treatments for tourette syndrome in children and young people – a systematic review. J Child Psychol Psychiatry. (2016) 57:988–1004. 10.1111/jcpp.12556 27132945

[27] Waller R Gilbody S. Barriers to the uptake of computerized cognitive behavioural therapy: a systematic review of the quantitative and qualitative evidence. Psychol Med. (2009) 39:705–12. 10.1017/S0033291708004224 18812006

[28] Fleming T Bavin L Lucassen M Stasiak K Hopkins S Merry S. Beyond the trial: Systematic review of real-world uptake and engagement with digital self-help interventions for depression, low mood, or anxiety. J Med Internet Res. (2018) 20:e199. 10.2196/jmir.9275 29875089 PMC6010835

[29] Bashiri A Ghazisaeedi M Shahmoradi L. The opportunities of virtual reality in the rehabilitation of children with attention deficit hyperactivity disorder: a literature review. Korean J Pediatr. (2017) 60:337–43. 10.3345/kjp.2017.60.11.337 29234356 PMC5725338

[30] Valmaggia LR Latif L Kempton MJ Rus-Calafell M. Virtual reality in the psychological treatment for mental health problems: an systematic review of recent evidence. Psychiatry Res. (2016) 236:189–95. 10.1016/j.psychres.2016.01.015 26795129

[31] Rodin A Fleetwood-Meade K Gilmour J Kasujja R Murphy T. Why don't children in Uganda have tics? a mixed-methods study of beliefs, knowledge, and attitudes of health professionals. Child Adolesc Ment Health. (2021) 26:47–53. 10.1111/camh.12370 32516519

[32] Khan K Hollis C Hall CL Murray E Davies EB Andrén P. Fidelity of delivery and contextual factors influencing children's level of engagement: process evaluation of the online remote behavioural intervention for tics (ORBIT) trial. J Med Internet Res. (2020) 23:e25470. 10.2196/25470 34152270 PMC8277316

[33] Khan K Hollis C Hall CL Davies EB Murray E Andrén P. Factors influencing the efficacy of an online behavioural intervention for children and young people with tics: process evaluation of a randomised controlled trial. J Behav Cogn Ther. (2022). 10.1016/j.jbct.2022.02.005

[34] Standing C Standing S McDermott ML Gururajan R Kiani Mavi R. The paradoxes of telehealth: a review of the literature 2000–2015. Syst Res Behav Sci. (2018) 35:90–101. 10.1002/sres.2442

[35] Greenhalgh T Wherton J Papoutsi C Lynch J Hughes G A'Court C. Beyond adoption: a new framework for theorizing and evaluating nonadoption, abandonment, and challenges to the scale-up, spread, and sustainability of health and care technologies. J Med Internet Res. (2017) 19:e367. 10.2196/jmir.8775 29092808 PMC5688245

[36] Lane-Fall MB Curran GM Beidas RS. Scoping implementation science for the beginner: locating yourself on the “subway line” of translational research. BMC Med Res Methodol. (2019) 19:1–5. 10.1186/s12874-019-0783-z 31253099 PMC6599376

[37] Baumeister H Reichler L Munzinger M Lin J. The impact of guidance on internet-based mental health interventions — a systematic review. Internet Interv. (2014) 1:205–15. 10.1016/j.invent.2014.08.003

[38] Andersson G Titov N Dear BF Rozental A Carlbring P. Internet-delivered psychological treatments: from innovation to implementation. World Psychiatry. (2019) 18:20–8. 10.1002/wps.20610 30600624 PMC6313242

[39] Mohr DC Riper H Schueller SM. A solution-focused research approach to achieve an implementable revolution in digital mental health. JAMA Psychiatry. (2018) 75:113–4. 10.1001/jamapsychiatry.2017.3838 29238805

[40] Graham AK Lattie EG Mohr DC. Experimental therapeutics for digital mental health. JAMA Psychiatry. (2019) 76:1223–4. 10.1001/jamapsychiatry.2019.2075 31433448 PMC7271442

[41] Vernon TW Koegel RL Dauterman H Stolen K. An early social engagement intervention for young children with autism and their parents. J Autism Dev Disord. (2012) 42:2702–17. 10.1007/s10803-012-1535-7 22527708 PMC3791600

[42] Haine-Schlagel R Walsh NE. A review of parent participation engagement in child and family mental health treatment. Clin Child Fam Psychol Rev. (2015) 18:133–50. 10.1007/s10567-015-0182-x 25726421 PMC4433419

[43] Brigden A Anderson E Linney C Morris R Parslow R Serafimova T. Digital behavior change interventions for younger children with chronic health conditions: systematic review. J Med Internet Res. (2020) 22:e16924. 10.2196/16924 32735227 PMC7428934

[44] Cuenca J Glazebrook C Kendall T Hedderly T Heyman I Jackson G. Perceptions of treatment for tics among young people with Tourette syndrome and their parents: a mixed methods study. BMC Psychiatry. (2015) 15:46. 10.1186/s12888-015-0430-0 25879205 PMC4359496

[45] Hall CL Sanderson C Brown BJ Andrén P Bennett S Chamberlain LR. Opportunities and challenges of delivering digital clinical trials: lessons learned from a randomised controlled trial of an online behavioural intervention for children and young people. Trials. (2020) 21:1–13. 10.1186/s13063-020-04902-1 33298127 PMC7724811

[46] Liao QV Fu WT. Age differences in credibility judgments of online health information. ACM Trans Comput Interact. (2014) 21:1–23. 10.1145/2534410

[47] Donkin L Glozier N. Motivators and motivations to persist with online psychological interventions: a qualitative study of treatment completers. J Med Int Res. (2012) 3:e2100. 10.2196/jmir.2100 22743581 PMC3414905

[48] Wantland DJ Portillo CJ Holzemer WL Slaughter R McGhee EM. The effectiveness of web-based vs. non-web-based interventions: a meta-analysis of behavioral change outcomes. J Med Int Res. (2004) 6:e116. 10.2196/jmir.6.4.e40 15631964 PMC1550624

[49] Brouwer W Kroeze W Crutzen R De Nooijer J De Vries NK Brug J. Which intervention characteristics are related to more exposure to internet-delivered healthy lifestyle promotion interventions? a systematic review. J Med Internet Res. (2011) 13:e1639. 10.2196/jmir.1639 21212045 PMC3221341

